# Agroecology landscapes

**DOI:** 10.1007/s10980-021-01248-0

**Published:** 2021-06-26

**Authors:** Ph. Jeanneret, S. Aviron, A. Alignier, C. Lavigne, J. Helfenstein, F. Herzog, S. Kay, S. Petit

**Affiliations:** 1 Department of Agroecology and Environment, Agroscope, 8046 Zurich, Switzerland; 2 UMR BAGAP, INRAE - Institut Agro-Agrocampus Ouest - ESA, 35042 Rennes, France; 3 UR PSH, INRAE, 84000 Avignon, France; 4 Agroécologie, AgroSup Dijon, INRAE, Univ. Bourgogne Franche-Comté, 21000 Dijon, France

**Keywords:** Agroforestry, Biodiversity, Ecosystem services, Landscape ecology, Pest regulation, Pollination

## Abstract

**Context:**

Agroecology combines agronomic and ecological concepts. It relies on the enhancement of biodiversity and related ecosystem services to support agricultural production. It is dependent on biological interactions for the design and management of agricultural systems in agricultural landscapes.

**Objectives:**

We review the role of landscape ecology to understand and promote biodiversity, pest regulation and crop pollination for the designing of “agroecology landscapes”. We illustrate the use of landscape ecological methods for supporting agroforestry systems as an example of agroecological development, and we propose pathways to implement agroecology at landscape scale.

**Methods:**

The state of the art of how landscape ecology contributes to agroecology development is summarized based on a literature review.

**Results:**

Agroecology requires thinking beyond the field scale to consider the positioning, quality and connectivity of fields and semi-natural habitats at larger spatial scales. The spatial and temporal organisation of semi-natural elements and the crop mosaic interact. Understanding this interaction is the pre-requisite for promoting patterns and mechanisms that foster biodiversity and ecosystem service provision. Promoting agroecological practices beyond individual farm borders can be rooted in a bottom-up approach from agroecological lighthouse farms to farm networks to amplify agroecology adoption at the landscape scale.

**Conclusions:**

Achieving agricultural landscapes composed of fields and farms following agroecological management requires understanding of biodiversity patterns, biological interactions and mechanisms that determine and boost ecosystem functioning to improve services at landscape scale, involving farmers in a bottom-up and context-specific approach.

## Introduction

There is broad agreement that agriculture needs to change rapidly and radically to sustainably meet future food demand (Roe et al. 2019; Cassman and Grassini 2020). Food demand is projected to increase 50% by 2050, so there is considerable pressure to improve productivity (Searchinger et al. 2018). However, productivity gains must be aligned with environmental goals, since intensive agriculture already has devastating effects on Earth system functioning through alterations of biogeochemical cycling, emission of greenhouse gases, and drastic loss of biodiversity (Zhang et al. 2007; Steffen et al. 2015). Also, as illustrated by the COVID-19 pandemic, agriculture must be resilient to unexpected shocks (Orden 2020; Worstell 2020). Agroecology has emerged as a scientific field, a set of agricultural practices, and a societal movement that holistically aims to transform agriculture to meet the above mentioned challenges (Altieri 1995; Wezel et al. 2009).

**Fig. 1:**
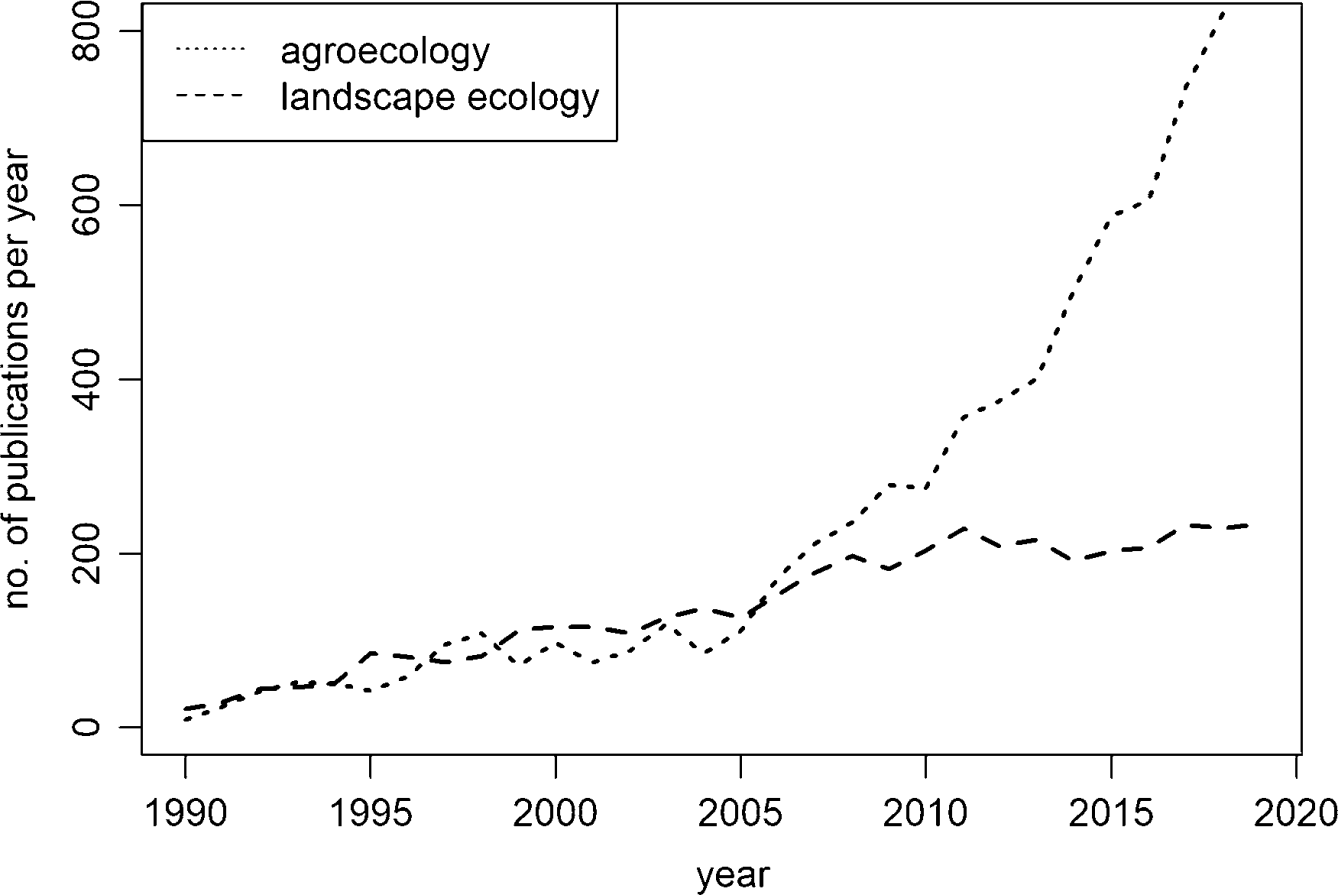
Growing number of scientific publications in the field of agroecology. The plot presents the number of publications listed in web of science published between 1990 and 2020, with the words “agroecolog*” or “agro-ecolog*” in the title, abstract or keywords. Search results for “landscape ecology” are shown as a reference

Agroecology uses ecological concepts and principles for the design and management of agricultural systems (Altieri 1995; Francis et al. 2003; Wezel et al. 2014). Agroecology started in the first half of the twentieth century as the overlay between agronomy and ecology, studying the ecology of crops and pests at the field-scale (Wezel et al. 2009; HLPE 2019). From these modest beginnings, the scientific discipline of agroecology has become broader, more interdisciplinary, and increasingly popular (Fig. 1). In reaction to the Green Revolution, farmers started to adopt “agroecological practices”, such as mulching, longer and more diversified crop rotations, and intercropping (Silici 2014; HLPE 2019). Since the 1980s, agroecology has evolved with the goal to provide an alternative to capital-intensive, industrial agriculture and empower smallholder farmers (Altieri and Toledo 2011). Agroecological movements today encompass a variety of environmentally-friendly farming efforts such as soil conservation practices, permaculture or organic agriculture (Pretty et al. 2018). However, agroecology also has strong roots in self-determination, food sovereignty and farmer’s rights (Silici 2014). For example, Zero Budget Natural Farming is a set of farming methods and a peasant movement spanning several million farmer families in India that focuses on reducing farmer debt by substituting chemical inputs with traditional knowledge and ecological processes (Khadse et al. 2018; Smith et al. 2020). In 2014, the Food and Agriculture Organisation of the United Nations (FAO) embraced agroecology as the way to holistically transform food systems, and has been active in promoting agroecology in global and national agricultural development strategies (FAO 2018; HLPE 2019).

Although agroecology has primarily focused on identifying sustainable farming practices at the field-scale, the discipline has increasingly considered the multiple embedded ecological and managerial scales at which processes take place, from the field to the landscape (Altieri 1999; Duru et al. 2015). This is because the fostering of many ecosystem functions delivering services required by an agroecological approach, such as crop pollination, pest regulation but also e.g. landscape aesthetics are dependent on processes occurring at the landscape scale (Tscharntke et al. 2005). In fact, the focus has shifted towards the question of redesigning agricultural landscapes that would deliver services to agriculture, an issue which has never been so high on the political and research agenda (Landis 2017). This can only be achieved by a concerted effort of ecologists, agronomists and social scientists, with the common goal of designing sustainable and resilient agricultural landscapes (DeFries and Rosenzweig 2010; Perfecto and Vandermeer 2010; Sayer et al. 2013; Bürgi et al. 2017).

For some key agricultural issues such as the prevention of crop disease spreading, the durability of crop resistance to pathogens, and the confinement of pest species with undesirable novel traits, the necessity for landscape scale approaches is obvious (Fabre et al. 2012; Václavík and Meentemeyer 2012). Integrated approaches combining agronomy and landscape ecology also allow addressing landscape scale drivers of organisms that were traditionally studied at the field scale (e.g. arable weeds, see Petit et al. 2013). Finally, agroecology promotes nature-based systems and as such relies on the enhancement of functional biodiversity to deliver services to agriculture, such as natural pest control and crop pollination. Promoting this functional biodiversity requires thinking beyond the field boundary, to consider the positioning, quality and connectivity of fields and semi-natural habitats at larger spatial scales (e.g. see Begg et al. 2017 for pest control services). Increasing connectivity of habitats for species conservation also has to consider potential unwanted effects on pest and invasive species spread (Maguire et al. 2015). Since its emergence as a discipline, landscape ecology has contributed to the development of holistic and transdisciplinary approaches aiming at delivering practical management solutions at a landscape scale (Naveh 2007; Wu 2013; Helfenstein et al. 2014; Gergel and Turner 2017). Such approaches have led to the development of frameworks and tools that fully acknowledge the complex ecological, economic and social interactions that drive the decision-making of stakeholders in the landscape, individually or collectively (Collins et al. 2011; Barnaud et al. 2018; Salliou et al. 2019).

Agricultural landscapes and their associated biodiversity are composed of a diversity of crop species and varieties, each combined with a large range of potential management practices, creating a complex mosaic of crop and off-crop habitats. Such landscapes are particularly dynamic, and changes occur in much shorter time scales than in most non-farmed landscapes. The organisms that populate these habitats have hugely diverse life-history strategies and as such exhibit contrasted responses to local and landscape management (Karp et al. 2018). For example, as they depend on crops, specialist pests are likely to exhibit less strong links to surrounding noncrop vegetation than generalist pests. On the contrary, specific natural enemies such as parasitoids will rely on semi-natural vegetation that provide pollen and nectar sources. The role of the spatio-temporal heterogeneity in shaping interaction networks between organisms in agricultural mosaics is highly relevant, as many of the expected services in agroecology result from these interactions, such as pest regulation and pollination (Massol and Petit 2013). Gaining a better understanding of the interplay between local and landscape scale management drivers of biocontrol, pollinators, or other species related to beneficial functions and services (Tscharntke et al. 2012), and of the general performance of agroecological farming systems (Smith et al. 2020) is a prerequisite for designing well-functioning agroecological landscapes.

In this article, we review how landscape ecological methods are applied to agroecology, with four foci:

- biodiversity conservation in agricultural landscapes as a foundation for agroecology
- the contribution of regulating ecosystem services such as pest control and pollination to agroecology
- agroforestry systems as an example for the promotion of agroecology
- implementing agroecological innovation in practice

Our review is mainly focused on arable landscapes, mostly in a European context. It does not cover rangeland landscapes, which in a broader sense, are agricultural landscapes as well. Yet, the agroecological mechanisms are fundamentally different between rangelands and arable landscapes, mostly due to the strong and frequent disturbance and the crop sequence in the latter. Furthermore, we are aware that biological interactions described in the next chapters are and will be affected by climate change. Here we rely on Altieri et al. (2015). In their review, Altieri et al. (2015) consider biodiversity at all levels as the key issue for making agricultural production systems sufficiently resilient with regard to climate change. In this context, our review in Chapter 2 of drivers and mechanisms involved in determining biodiversity conditions and regulation services in agricultural landscapes can be considered as a foundation for establishing interventions to adapt to climate change.

## Biodiversity conservation in agricultural landscapes as a foundation for agroecology

Agricultural intensification has led to the decline or disappearance of many species in agricultural landscapes, which have resulted in changes in the structure of communities, in biological interactions and trophic networks, as well as in the functioning of agroecosystems (Matson et al. 1997; Tilman et al. 2001). Thus, the conservation of species diversity (i.e. species richness and relative abundance of species in communities) in agricultural landscapes is crucial not only to stop the overall decline of biodiversity but also to ensure the maintenance of multiple biological functions (Manning et al. 2018), some of them delivering important services for agroecosystems (Liere et al. 2017; Tamburini et al. 2020). In turn, these services such as those supplied by soil organisms, regulation processes (pollination, pest control) are the foundation of agroecology (see Chapter 3). Understanding the mechanisms responsible for changes in species diversity and improving conservation and functioning requires taking a landscape approach (Seibold et al. 2019). Species interact with landscape properties in various ways and several ecological processes are related to these interactions, which in turn act on the distribution and dynamics of species, and the structuring of communities (Dunning et al. 1992; Tscharntke et al. 2012): (a) complementation/supplementation processes, where species use non-substitutable/substitutable resources from different habitats; (b) neighboring or mass effects, reflecting the strong influence on a local population or community of direct adjacent habitats, through spillover processes at habitat edges, and (c) concentration/dilution processes, where the decrease or increase of habitat area at the landscape scale results in a strong increase ('concentration') or decrease ('dilution') in species abundance in habitats; (d) source-sink relationships, where habitats with high population levels act as sources of migrants for ones with low population level ('sink'). At the metacommunity level, landscape properties determine species dispersal between local communities, which strongly contribute to the spatial structuring and persistence of metacommunities at large spatial scales (Chase 2005). Some of these concepts are illustrated below.

### The role of landscape properties for the structuring of communities and metacommunities

In agricultural landscapes, ecological studies have initially investigated the role of landscape structure on animal and plant communities following the habitat-matrix paradigm, where semi-natural elements (e.g. woodlots, hedgerows or herbaceous strips) are imbedded in a homogenous and hostile agricultural matrix (e.g. Billeter et al. 2008). Overall, a high share and degree of spatial connectivity of semi-natural elements were found to increase the diversity and abundance of a wide range of taxa in agricultural landscapes, including butterflies, carabid beetles, birds and plants (e.g. Billeter et al. 2008; Bailey et al. 2010; Duflot et al. 2018). Being ecological corridors, linear semi-natural elements enable movements (dispersal) of individuals and genes between habitat patches and across the landscape (e.g. Thiele et al. 2018). Because such dispersal events (colonization/emigration) enable to link local communities, habitat amount and connectivity are also key drivers of metacommunity structuring and persistence in the landscape (Wilson 1992; Leibold et al. 2004; Chase 2005; Thompson et al. 2017).

**Fig. 2:**
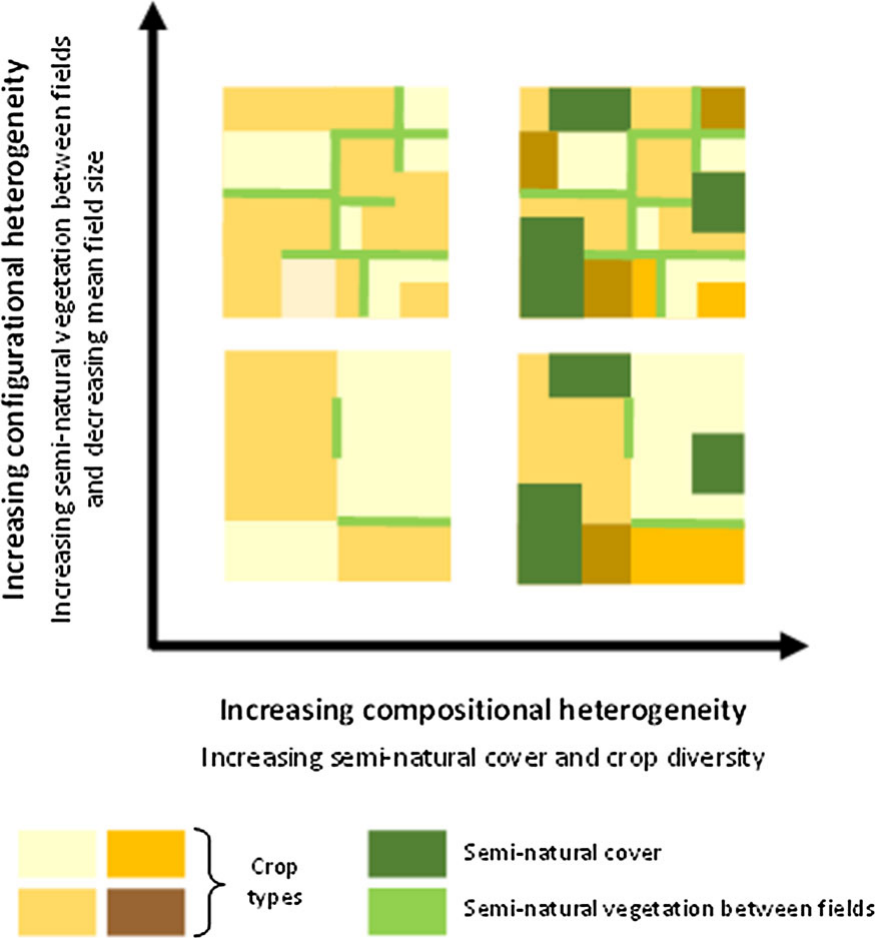
Illustration of the two components of landscape heterogeneity, composition (horizontal axis) and configuration (vertical axis). In agricultural landscapes, compositional and configurational heterogeneity depend on the amount, diversity and configuration of both semi-natural vegetation and crops. (adapted from Fahrig et al. 2011)

There has been growing awareness that the heterogeneity of the entire mosaic, not only of semi-natural habitats, is a major driver of species diversity and ecological processes in agricultural landscapes (Benton et al. 2003; Polis et al. 2004; Lovett et al. 2005; Sirami et al. 2019). Landscape heterogeneity, defined as the composition (diversity, quality and amount or surface area of habitats) and configuration (spatial arrangement of habitats) of a landscape (Fig. 2; Fahrig et al. 2011; Sirami et al. 2019), has thus become a central concept in landscape ecology. At the community and metacommunity levels, landscape heterogeneity is expected to act as an ecological filter of species and ecological traits (Gámez-Virués et al. 2015), by determining the diversity of habitats for species (composition) and by impacting species dispersal between habitat patches (configuration) (Ricketts et al. 2008; Fahrig et al., 2011). Investigating the role of agricultural landscape heterogeneity, recent research found that mean field size and crop diversity are important drivers of the species diversity of various organisms including birds, carabid beetles, spiders, pollinating insects and plants (Fahrig et al. 2015; Hass et al. 2018; Sirami et al. 2019; Alignier et al. 2020). Increasing compositional crop heterogeneity (namely crop diversity) may increase overall species richness if species are specialists of different crop types by creating bigger resources availability (Benton et al. 2003; Fahrig et al. 2011). It may also enhance species that require multiple resources for their life cycle (i.e. resource complementation, Dunning et al. 1992; Fahrig et al. 2011). Increasing configurational crop heterogeneity by decreasing mean field size may facilitate cross-habitat spillover, i.e. species movement between crop and adjacent semi-natural features (e.g. Henckel et al. 2015). Recent work showed that mean field size contributes more strongly to the diversity of pollinating insects and vascular plants in arable fields than an increasing amount of semi-natural elements (Hass et al. 2018; Alignier et al. 2020). Landscape heterogeneity has also been highlighted as an important driver of dispersal processes involved in metacommunity structuring and stability (Jacobson and Peres-Neto 2010; Ryberg and Fitzgerald 2016).

Another way to describe landscape heterogeneity consists in considering the heterogeneity resulting from the spatial distribution of farming practices (Vasseur et al. 2013). However, exploring the role of landscape heterogeneity related to the crop mosaic remains a methodological challenge, especially regarding the description and mapping of farming practices at large spatial scales.

### The importance of landscape dynamics to explain biodiversity patterns in agricultural landscapes

Agricultural landscapes are not only heterogeneous in space but are also dynamic within a year (in relation with crop phenology and successive management operations), over several years (in relation with crop rotation), over decades (e.g. reallotment affecting the size and the shape of cropping and semi-natural areas) or even over centuries (land cover changes from rangeland to arable land or vice-versa). Such dynamics can strongly affect the current distribution patterns of species. For instance, landscape changes over short periods of crop succession and intra-annual resource variability are important drivers for population dynamics and species diversity (Wimberly 2006; Thies et al. 2008; Baselga et al. 2015). In contrast, other ecological studies focusing on long-term land use changes and their effects on plants (Lindborg and Eriksson 2004), vertebrates (Metzger et al. 2009) and invertebrates such as carabid beetles (e.g. Petit and Burel 1998; Alignier and Aviron 2017) have shown that the present distribution and diversity of species in agricultural landscapes are better explained by past landscape properties (one to 5–7 decades) than current ones. Further back in time, it has been shown that Roman agricultural systems continue to drive plant species diversity in French forests, even two millennia after abandonment (Dambrine et al. 2007), whereas the legacy of pre-Columbian land use continues to shape Amazonian species diversity and distribution (Maezumi et al. 2018). This suggests that landscape-level ecological assessments should not only be extrapolated from results based on static maps, but need to consider legacy effects as well (Wimberly 2006).

### Responses of biodiversity to the intensity of farming systems in heterogeneous landscapes

Drivers of biodiversity patterns and associated ecosystem functions occur at various spatial scales from global biogeographical conditions to local microhabitat quality. In agricultural landscapes, farming activities are driven by processes operating at several levels from regional agricultural policies to individual farming practices in the field. The use of a holistic and hierarchical approach, based upon the hierarchy theory, has thus been advocated to relate farming activities and responses of agroecological systems (Baudry et al. 2000; Turner et al. 2001). According to hierarchy theory, complex systems have a vertical and nested structure in which lower-level components are contained and constrained by upper-level components (Wu and David 2002). By consequence, upper-level drivers are supposed to have more important effects than lower-level drivers. Accordingly, landscape properties are expected to have a higher effect on species diversity than local factors such as farming practices. Although several studies have highlighted higher impacts of landscape heterogeneity on species diversity compared with local farming practices or farming systems (Bengtsson et al. 2005; Trichard et al. 2013; Martin et al. 2020), others have found contradictory results (e.g. Holzschuh et al. 2007). Despite these inconsistencies, there is now empirical evidence of strong interactions between the response of animal and plant communities to farming intensity at field or farm levels and landscape heterogeneity. Contrasting assumptions regarding these interactions have been proposed in the literature (Concepción et al. 2008). The differences in species diversity levels between low and high intensity farming (e.g. fields under organic farming or agri-environmental schemes *versus* conventional farming) were found to be (a) independent of landscape heterogeneity (no relationships between factors), (b) less important in heterogeneous landscapes (effects of farming intensity on biodiversity are compensated by landscape heterogeneity), (c) less important in homogeneous landscapes (enhancement effect of landscape heterogeneity on the effects of farming intensity reduction on biodiversity), or (d) more important in landscapes with intermediate heterogeneity (enhancement effect of landscape heterogeneity in simple landscapes and compensation effect of landscape heterogeneity in complex landscapes; e.g. Concepción et al. 2008; Brittain et al. 2010; Batáry et al. 2011) Despite the observed variability in the type of interactions between local farming practices and landscapes properties, several authors have concluded that agri-environment schemes might be the most effective for improving biodiversity in landscapes with intermediate heterogeneity, but inefficient in simple or highly heterogeneous landscapes (Tscharntke et al. 2005; Concepción et al. 2008). These studies underline the need to integrate multiple scales (field, farm, landscape) when analyzing the drivers of biodiversity patterns and associated ecological functions in agricultural landscapes (Gonthier et al. 2014).

Enhancing the conservation of farmland species diversity requires a landscape perspective. The implementation of wildlife friendly practices at farm level, such as increasing the amount of suitable habitats for species, has resulted in beneficial effects on biodiversity (e.g. Aviron et al. 2009, 2011). However, how to coordinate such practices amongst farms to enhance habitat connectivity or landscape heterogeneity remains a critical challenge as well as to promote particular functions and services (see Chapters 3 and 5). Interdisciplinary research, integrating social sciences, might allow to better understand the conditions that enhance or impede farmer collaboration in that context (Steingröver et al. 2010).

## The contribution of the regulating ecosystem services pest control and pollination to agroecological landscapes

### Landscape ecology and the agroecological control of pests

The promotion of nature-based control of pests in agriculture is a major pillar of agroecology, as it supports natural ecosystem processes to reduce pesticide use by complexifying ecological networks at all scales (Bohan et al. 2013). Conservation biological control in particular, aims to improve the management of pests by supporting populations of natural enemies that are already present in the agroecosystem, and by promoting their effectiveness at reducing pest populations. As for plant and animal communities in general (see Chapter 2), a number of studies have thus focused on the impact of landscape heterogeneity on the abundance of pests and their suppression by natural enemies with the aim to promote conservation biological control (Begg et al. 2017). More recently, some studies have explored the potential of the spatial expansion of agroecology at a landscape scale to enhance pest control (Petit et al. 2020). Such studies typically investigate how natural enemy abundance or diversity, predation and parasitism, or pest abundance in fields depend on the surrounding landscape (e.g. Coudrain et al. 2014). Both abundance and species diversity of natural enemies are expected to enhance the natural control of pests (Dainese et al. 2019).

#### Importance of semi-natural elements for natural enemies

Landscape composition is usually defined by the amount of semi-natural elements, possibly split in different categories (e.g. woody, grassland) and by the area of cultivated land (also possibly broadly categorized, e.g. annual versus perennial). The role of semi-natural elements has been particularly highlighted as they provide food or shelter for many natural enemy species (Landis et al. 2000; Duelli and Obrist 2003; Holland et al. 2016). However, they can also provide resources for pests (e.g. aphids, see Alignier et al. 2014), so that the balance in terms of pest control is not always favourable (Tscharntke et al. 2016). Several meta-analyses indicate that cultivated plots located in landscapes with a higher proportion of semi-natural elements host more natural enemies but this increase does not always translate into decreased pest abundance (Chaplin-Kramer et al. 2011; Veres et al. 2013; Karp et al. 2018). The resources provided by semi-natural habitats can be broadly divided into three categories: food, alternate or secondary hosts and overwintering sites. Depending on pest or natural enemy species, these resources are found in different habitats. In an effort to have a more functional approach, the effect of particular categories of semi-natural habitats has therefore been investigated. A canopy dwelling spider in orchards, for example, was more abundant when the proportion of woody elements increased in the landscape (Lefebvre et al. 2016). In contrast, the abundance of carabid beetles found in arable crops responded to the amount of grassland in the surrounding landscape (Labruyere et al. 2016). More specific studies of species’ needs may also guide the choice of investigated land uses. For example, the abundance of adult hoverflies correlated positively with the amount in the landscape of habitats specifically hosting the flowering species on which they feed (Vialatte et al. 2017).

#### Investigating the role of the crop mosaic and farming systems

In addition to landscape composition (amount of habitat), landscape ecologists and agronomists have more recently investigated the impact of the crop mosaic and its heterogeneity on pest suppression by natural enemies, i.e. the role played by the crop mosaic configuration (e.g. number of patches per landscape, patch size or interpatch connectivity, Fahrig et al. 2011) combined with crop mosaic composition (see Chapter 2). For instance, it has been shown that the effect of crop mosaic heterogeneity on pest control may depend on the amount of semi-natural habitat (e.g. Bosem Baillod et al. 2017). As for the configuration component, a decrease in average field size and increase in interface length is expected to facilitate movements between semi-natural habitats and cultivated fields, and among cultivated fields. Consistent with this expectation, in an analysis of 49 studies, edge length had a positive effect on pest control (measured as pest predation or parasitism) only when the proportion of landscape grown with arable crop was below 40% (Martin et al. 2019). In another review (Haan et al. 2020), 33 recent studies provide evidence of landscape configuration effects on pest suppression. Here it was emphasized that agricultural landscapes with small fields and/or large edge lengths can enhance natural enemies, particularly those overwintering outside cultivated fields, but direction and strength of effects on pest suppression were context dependent.

Studies on the effects of crop management at landscape scale have mainly focused on the impact of organic or conventional farming systems. The abundance of pests was not affected (Ricci et al. 2009 for apple orchards; Muneret et al. 2018 for vineyards) or decreased (Gosme et al. 2012 for arable crops) in landscapes with increased proportion of organic farming, whereas weed diversity, but not abundance, was increased (Petit et al. 2016). Furthermore in vineyards, an increasing proportion of organic farming in landscapes generally had no effect or increased the abundance of natural enemies (Diekotter et al. 2010; Puech et al. 2015; Djoudi et al. 2018). When present, this weak or beneficial effect of landscape scale organic farming on pest control despite reduced pesticide efficiency in comparison to conventional farming might result from decreased natural pest control in landscapes dominated by conventional crops. For example, the abundance of codling moth larvae, the main apple pest, but also of its parasitoids and the predation of codling moth sentinel eggs all decreased when the area grown with conventional orchards increased in an intensive apple growing landscape (Ricci et al. 2009; Maalouly et al. 2013; Monteiro et al. 2013). Similarly, aphid predation decreased with increasing landscape scale pesticide use in arable crops (Meehan et al. 2011). These results underline the importance of considering landscape scale crop management in landscape ecology approaches for pest control and call for investigations on other agricultural practices (e.g. no till, irrigation, insect-proof nets).

#### Landscape temporal dynamics

Few studies have focused on the impact of past landscape structure on pests or pest enemies, although legacy effects are expected to be strong (see above). Landscape composition changes across time mainly due to crop rotation. Regarding pests that are specialized on a particular crop, this may result in a variation of the available amount of resources across years in relation with the area of the crop over the landscape. This dynamic creates dilution/ concentration of pests in the crop. Variations in oilseed rape crop area from one year to the next for example appeared to be a good predictor of the in-field abundance of pollen beetle pests (*Meligethes aeneus*) the following year (Schneider et al. 2015) as pollen beetles may overwinter in former oilseed rape fields (Sutter et al. 2018). Further, this dilution/concentration effect may transfer to higher trophic levels. An expansion of oilseed rape area from one year to the next, for example, led to decreased parasitism of *M.aeneus* the second year (Thies et al. 2008). Similar processes may affect the distribution of less specialized species of pests or predators, in particular if they need a particular sequence of crops within a year or between years to cope with changes in resources needed or habitat suitability for their different life-stages. The aphid pest *Sitobion avenae* was for example shown to complete its life cycle by dispersing from ripe maize to newly planted wheat in autumn (Vialatte et al. 2006). In a five year study, the abundance of dominant carabid beetle species was also shown to increase with the temporal heterogeneity of the landscape, measured as the change in crop diversity over years (Bertrand et al. 2016).

#### Sources of variability

Although broad trends emerge regarding landscape effects on biological control of pests, there is a large variability among studies. Some of this variability may be due to methodological issues (see Chapter 2). It may also result from the species’ ecology, some species making little use of semi-natural habitats and finding resources in cultivated fields (Tscharntke et al. 2016). A study of parasitoids of cereal aphids for example demonstrated a specialization of parasitoid species on aphid species from either crops or uncultivated plants, so that semi-natural habitats were not a reservoir of parasitoids for pest control (Derocles et al. 2014). Some parasitoids (e.g. Navasse et al. 2018) or carabid species (e.g. Petit et al. 2017) may specialize on crop areas. The intensity of local farming practices in the field is a further source of variability (Ricci et al., 2019). In the case of very high intensity practices, colonization of the cultivated plots would be impeded because the colonizing individuals would not survive (Tscharntke et al. 2016); in the case of very low intensity plots, on the contrary, species finding most resources within the field would not need to disperse to other fields or semi-natural habitats. Such examples of interactions were reported by Tamburini et al. (2016) on parasitism and predation of aphids by vegetation-dwelling organisms, and by Petit et al. (2017) on seed-eating carabid abundance and weed seed predation in cereal fields.

### Landscape ecology and crop pollination

As with natural pest control, the pollination of crops is an indispensable service to the persistence of agroecological systems, perhaps even more so than for conventional systems, as pollination should be optimal to compensate for other possible sources of production losses such as those induced by pests (Kleijn et al. 2015; see Sutter and Albrecht 2016 for an example of interplay between pest control and pollination). Previous reviews indicate that *c.* 35% of the crop production volume and *c.* 70% of major global crops rely on animal pollination (Klein et al. 2007; Aizen et al. 2009). The majority of food crops require pollination to set fruit with the honeybee being the main pollinating insect. However, wild pollinators are also a vital part of agricultural systems. In more than 40 important crops grown worldwide, wild pollinators improved pollination efficiency, doubling the fruit set compared to honeybees (Garibaldi et al. 2013). Beside domestic and wild bees, non-bee pollinators including flies, beetles, moths, butterflies, wasps are shown to be efficient pollinators, providing 39% of visits to crop flowers (Rader et al. 2016).

The reported large scale parallel declines of plants and wild pollinators are alarming, as they could imperil future ecosystem stability and food security (Biesmeijer et al. 2006; Tylianakis 2013; IPBES 2016). Landscape changes resulting from agricultural intensification and leading to habitat loss and fragmentation are a major threat to pollination services (Kremen et al. 2002; Steffan-Dewenter and Westphal 2008; Winfree and Kremen 2009).

#### Importance of semi-natural elements for pollinators

Over the past decades, there has been a wealth of research testing the effects of landscape attributes (e.g. habitat amount, connectivity, patch size) on pollinators and pollination (Steffan-Dewenter and Westphal 2008; Martin et al. 2019; Joseph et al. 2020). These studies mainly focused on the semi-natural habitats, which provide food as well as sites for nesting and reproduction (Gathmann and Tscharntke 2002; Westphal et al. 2003). Loss of semi-natural habitats such as forests and grasslands has been shown to have strong negative effects on plant and pollinator abundances (Steffan-Dewenter and Schiele 2008; Winfree et al. 2009; Proesmans et al. 2019) resulting in lower pollen availability, fewer numbers of flower–visiting bees (Steffan-Dewenter et al. 2001) and fewer vectors to move pollen through the landscape (Winfree and Kremen 2009) with consequences on wild plant and crop pollination (Ricketts et al. 2008). Similarly, fragmentation and isolation of semi-natural habitats have generally negative effects on pollination and on fruit and seed production (e.g. Aguilar et al. 2006; Garibaldi et al. 2011; Schüepp et al. 2013). By limiting the survival and movement of pollinators, those landscape processes may result in species extinctions and could lead to disruption of plant-pollinator interactions with unpredictable consequences for the maintenance of biodiversity and environmental services (e.g. Steffan-Dewenter and Tscharntke 1999).

#### The role of the crop mosaic

Pollinators may also benefit from agricultural activities taking place in the agricultural matrix, still often considered as “hostile”, such as ground-nesting bees that use disturbed areas for nesting or pollinators foraging pollen-rich crop fields (Klein et al. 2007; Westphal et al. 2003). However, as with certain pest control agents, the mobility of pollinators might be affected by the crop mosaic and its heterogeneity as it is by the loss and isolation of the semi-natural habitats. Studies focusing on the effects of the composition of the crop mosaic (i.e. diversity of crop cover types) have only recently started, and the outcomes have been inconsistent. Bee abundances and species richness have been shown to decline (Hass et al. 2018; Martin et al. 2020), increase (Sirami et al. 2019; Raderschall et al. 2020) or to be unaffected (Fahrig et al. 2015; Aguilera et al. 2020) by increasing landscape crop diversity. The studies of the effect of crop diversity in the mosaic on pollination service showed no effect of crop diversity on the seed-set of phytometer plants (Hass et al. 2018) nor the seed weight per plant (Raderschall et al. 2020). Similarly, the few available studies investigating the impact of configuration of the crop mosaic (i.e. the shape and spatial arrangement of crop fields, which are measured as the mean size of fields or edge density for instance) show contrasting results. Most of them found no or weak evidence that crop configuration influences pollinator communities (Kennedy et al. 2013; Steckel et al. 2014).

Such discrepancy in results might be explained by the major role played by the identity of the crop sown, namely crop species or variety. For instance, high proportions of mass-flowering crops (e.g. oilseed rape, sunflower, legumes) may attract pollinators by providing ample resources, though limited in time (Westphal et al. 2003). Farming practices may also impact the distribution of pollinators in agricultural fields independently of crop identity. For instance, the improved habitat quality in crops under suitable farming management might boost pollinator abundance, with organisms spilling over to nearby fields (Tscharntke et al. 2012; Kennedy et al. 2013). Further reasons that may explain highly variable ecological responses of pollinator groups to landscape features, may be related to contrasting flower–visitor behavior, dispersal abilities, or foraging distances (Steffan-Dewenter et al. 2001; Hadley and Betts 2012).

Deliberate manipulation of plant communities at both local and landscape scale is currently the best understood management tool for conserving pollination services within agricultural landscapes (Kremen et al. 2007). Mass-flowering crops as well as flower-rich semi-natural elements can help support pollinators. Though less studied, in-field plant diversity or “weeds” may provide additional resources especially after the blooming period of mass-flowering crops (Bretagnolle and Gaba 2015; Lichtenberg et al. 2017). Knowledge gaps relate to how landscapes facilitate pollinator movements (connectivity) between crop areas and nesting and foraging habitats. Integrated landscape management must focus on both structural and functional connectivity between environments. In this way, it would be possible to ensure effective pollen flow and, consequently, fruit and seed production.

Biological pest control and crop pollination are two major functions for the agroecosystem. As we have shown, understanding their functioning and promotion requires a landscape scale approach. Still, both functions are also affected by the farming system they deliver services to. The next chapter focuses on agroforestry as an example of an agroecological system, and how landscape ecological approaches support its development.

## Landscape ecological methods to promote agroecological landscapes: the example of temperate agroforestry

Taking agroforestry as an example, here we illustrate how landscape ecological research supports the development of modern, temperate agroecological farming. Agroforestry combines the use of trees with annual crops or fodder plants and possibly with livestock rearing on the same piece of land (Somarriba 1992). The components interact and—if they are well chosen and arranged—build up synergies, which leads to higher resilience and allows to maintain long-term productivity (Burgess and Rosati 2018). Agroforestry is now increasingly discussed as an alternative to conventional agriculture, since recent studies highlight that agroforestry “relicts” enhance biodiversity and provide additional ecosystem services (e.g. Moreno et al. 2018; Pantera et al. 2018). The mosaic of annual and perennial crops, open managed areas versus permanent patches as well as the multiple vegetation layers create various nesting, foraging and living habitats for flora and fauna (Moreno et al. 2016; Lecq et al. 2017; Bailey et al. 2010) Consequently, most of the traditional systems are recognized as “high nature value farmland” e.g. fruit orchards (Herzog 1998), Dehesa/Montada (Moreno and Pulido 2009), bocage (Lecq et al. 2017); preserved as valuable habitats within the European Natura 2000 network (EEA 2015) and appreciated by society e.g. as “Landscapes and Natural Monuments of National Importance” (Bundesrat 2010). Maes et al. (2012) showed that areas with favorable conservation status maintain and improve biodiversity and at the same time (regulating and cultural) ecosystem service provision. In line with this, Alam et al. (2014) and Torralba et al. (2016) compiled ecosystem services linked to agroforestry systems such as nutrient cycling, water, air and soil quality, pollination, biological control of pests, windbreak, timber and agricultural production, and climate regulation. Both—biodiversity conservation and ecosystem service provision—are recognized by policy and society, at least partly, and consequently traditional agroforestry systems are supported by government payments (Santiago-Freijanes et al. 2018).

The question now is, if modern agroforestry systems (Nerlich et al. 2013), which are practicable with modern farming technology and mechanization, still provide ecosystem services and comparatively higher levels of farmland biodiversity than agricultural monocropping. Tool-boxes were developed to evaluate ecosystem services provided by agroforestry systems, first at the field and farm scale (Palma et al. 2007; Tsonkova et al. 2014) and then at the landscape scale (Kay et al. 2019a). They combine well established methods for evaluating biodiversity, soil conservation and water fluxes with specifically developed process models that account for the interaction between trees and crops (García de Jalón et al. 2018) and can also accommodate life cycle assessment (Crous-Duran et al. 2019b) and approaches for evaluating cultural ecosystem services (Fagerholm et al. 2019). The toolboxes allow comparing ecosystem service provision (including productivity; Kay et al. 2019a) or to simulate the consequences of an uptake of agroforestry systems in scenario studies (Crous-Duran et al. 2019a).

Acknowledging the environmental benefits provided by agroforestry systems encourages a widespread implementation. While farmers and administrators operate at the plot and farm scale, policy makers have a more strategic perspective and require information at larger spatial scales, i.e. national or continental. To inform about the potential impact of agroforestry, Reisner et al. (2007) identified regions of European arable land, where environmental risks by intensive farming accumulated. Implementing agroforestry systems could contribute to soil protection on 4%, mitigate nitrate leaching on 18% and increase landscape diversity on 32% of European arable land. Kay et al. (2019b) broadened the approach to include animal based silvopastural agroforestry systems and to evaluate additional ecosystem services. They identified 64 regionally adapted agroforestry systems and concluded that transforming only 9% of the European agricultural landscapes to agroforestry would allow to compensate for up to 43% of the greenhouse gas equivalents attributed to the European agricultural sector. In the United States, Wolz et al. (2018) identified regions, where agroforestry would be more profitable and ecological friendly than the currently widespread maize-soybean rotation that degrades many ecological functions. Mattia et al. (2018) mapped suitable regions for silvopastural agroforestry in Illinois and Ahmad and Goparaju (2017) summarized several approaches to land suitability mapping for agroforestry, with an application to India.

Mainstreaming the implementation of agroforestry as an agroecological system requires first to identify adequate regions. Besides geomorphological and farming type characteristics, socio-economical features and context-specific conditions need also to be taken into consideration. The next chapter proposes pathways to implement agroecology at farm and landscape level.

## Agroecology landscapes into practice

The farm scale is a lever for the implementation of agroecological principles, but also a hurdle when it comes to developing environmentally-friendly farming at the landscape scale. Farms are legal/economic units that are rented/owned by the farmer and consist of various land units. Farmers decide on management practices, which makes them the most important decision-maker on crops grown, their spatial and temporal arrangement within a crop rotation or as perennial plantation. However, individual farms may be intermingled with plots of land owned by other farmers not performing agroecology, or with land that has no agricultural function (Herzog et al. 2017). The non-contiguous nature of many farms means that the spatial arrangement of the habitats of an individual, non-consolidated farm has no ecological integrity (e.g. Cumming et al. 2006). Thus, individual farmers are limited in their action space to influence ecosystem functions operating at the landscape scale.

While there are many challenges dealing with a landscape approach to agroecology, there are also many action levers. Indeed, agroecology depends on context-specific knowledge, and practices need tailoring to fit the local to regional, environmental, economic, social, cultural and political context. Mainstreaming agroecology requires blending traditional and local knowledge, and eco-technological innovations. Thus, a possible strategy to circumvent the incompatibility of decision making and ecologically relevant scales is the adoption of a multi-actor, bottom-up, and small to medium scale approach. Farms can be organized in regional cores of agroecological, interlocking farms, so called *“agroecological lighthouses”,* following concepts of Nicholls and Altieri (2018).

Such an approach requires a co-creation process followed by farmers, scientists, advisers, enterprises, NGOs, etc. after a first initiative of one or the other group. The goal is that individual farms would benefit from being in the vicinity of other agroecological farms. A participatory approach allows knowledge to flow between different stakeholders.

This requires the following, though not exclusive, steps:

1. Develop *scientific foundation* of agroecological management that will be the cornerstone of the transdisciplinary, participatory and multi-actor conceptual framework. The scientific foundation needs to be evidence-based, to reflect effective management practices and be praxis-oriented. However, the co-creation process should also stimulate ideas of new practices coming from the farmers and the advisors to be tested. This should be done with full implication of scientific staffs for designing experiments.
2. *Identify regional cores of farms to become agroecological lighthouses* and establish a *network* committed to testing and implementing agroecological management strategies. Criteria for identification of those farms encompass the willingness of the farmer to participate to a multi-actor decision-making process for innovation, to engage in a process of changing usual practices, and to monitor and report on the changes.
3. *Identify farmers’ needs and constraints* regarding the adoption of agroecological management methods, in the framework of multi-actor groups, following the Agricultural Knowledge and Information System (AKIS) strategy (Schut et al. 2014). For instance, the delivery of ecosystem services focusing on soil fertility, natural pest control and crop pollination should help knowledge transfer and adoption. But particular attention has still to be put on farmers’ perception of the process in order to reinforce their pride of participating (Dessein and Nevens 2007). These steps are key to provide solutions for the removal of barriers limiting the translation of agroecological management into practice.
4. *Design and implement novel functional agrobiodiversity management strategies* on farms.
5. Provide solutions for the *removal of barriers limiting the translation of agroecological management into practice*. These barriers may be ecological, economic, technological, societal, and psychological barriers.
6. *Farm-level observatories* committed to monitoring progress made in the farm networks regarding the implementation of agroecological management strategies.
7. *Assess the performance* of agroecological management strategies on the environmental, economic and social sustainability of farms. Design the implementation of new practices and strategies in the existing networks.
8. *Disseminate best agroecological management strategies* to all relevant stakeholders and interested parties within the farm networks and beyond.
9. *Build new cores of farm networks* based on lessons learned from agroecology lighthouses.

Box 1

Example 1: System approach of best practices based on agroecology to reduce pesticide use in arable crops of farm networks in Switzerland (www.pestired.ch).

A system approach for crop rotations has been launched in Switzerland aimed at reducing the use of chemical pesticides by implementing agroecological measures in commercial farms (Wirth et al. 2020). Following the steps described above, 20 agroecological action levers were proposed from scientists to farmers, such as mixed cropping, mechanical weed control, wildflower strip implementation, undersowing, etc. In 2019, participating farmers decided which practices to implement in one single field but during a whole 6-year crop rotation. First observations after co-innovation workshops (70 farms) show that farmers are willing to reduce chemical pesticide use for the sake of human and environmental health, but would be reluctant to participate in whole-farm setups. The most important barriers are a) risk of yield and quality reduction, b) insufficient market for niche crops, c) insufficient knowledge on particular practices, d) intrinsic resistance to changes. After one year of implementation, resistance to changes has been smoothed by regular exchanges between the scientist, the advisors and the farmers. Knowledge on particular practices has also improved through the same process, yet not all innovative practices have been implemented. In all of that, exchanges amongst farmers have played a crucial role. This is a main unlocking lever. With regard to the potential yield reduction, data analysis is ongoing. There have been no niche crops sown in any of the participating farms. The question of implementing niche crops concerns the whole value chain of agricultural production and will not be solved in that particular project. Intermediary results will be reported along the course of the project, though effects of agricultural measures will be best analysed by the end of the crop rotation (2025). 
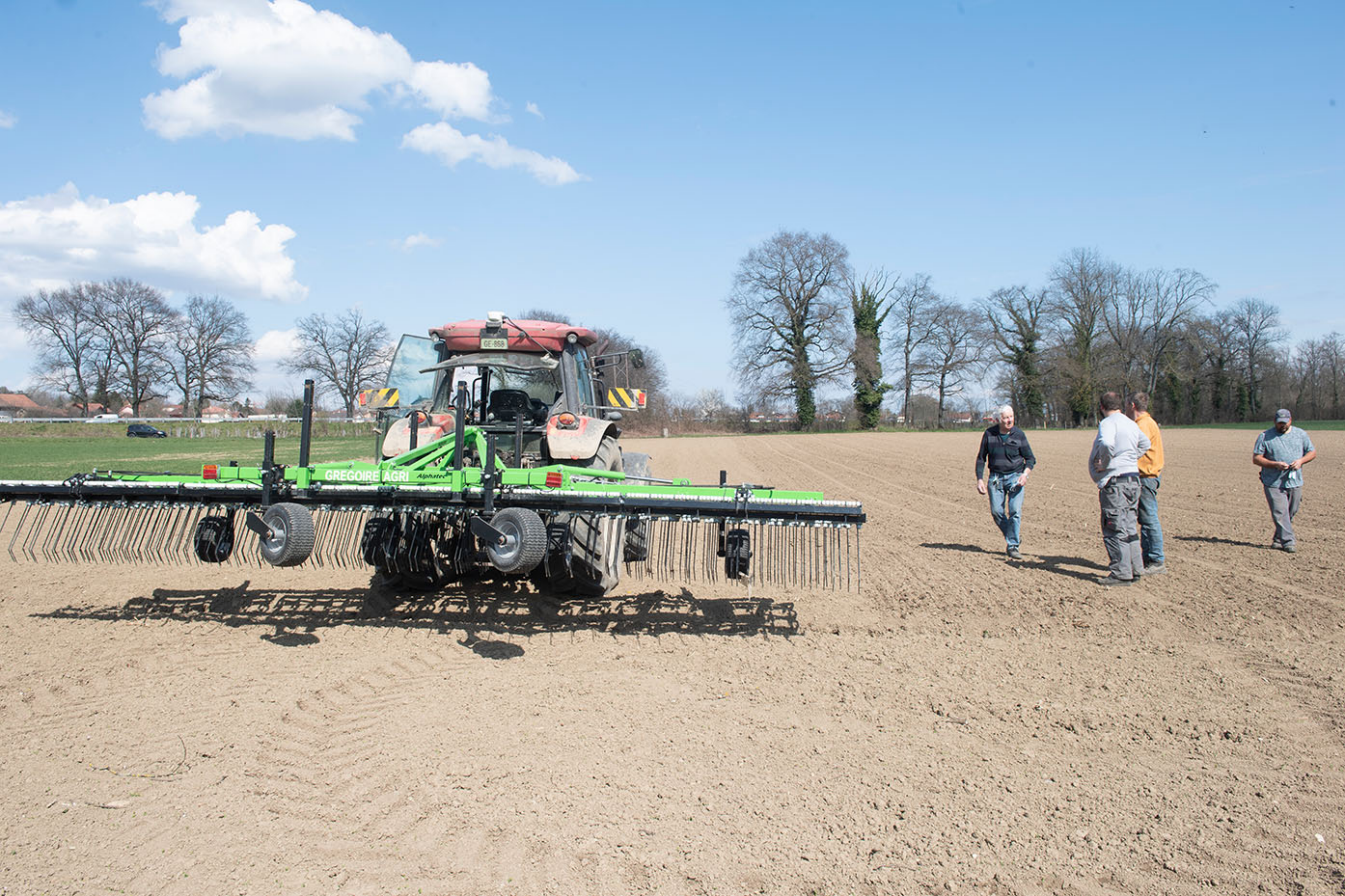

Example 2: Experimental and commercial farm partnership to foster biodiversity-based agroecological transition in farming wetlands in France (Transi’Marsh https://www.herbea.org/fr/news/8068/transi%27marsh:-suivi-de-la-transition-agroécologique-dans-le-marais-de-st-laurent-de-la-prée).

In an effort to make an agroecological transition of an experimental farm in a wetland region (INRAE, St Laurent de la Prée, Nouvelle Aquitaine), farmers of the region have been interviewed to identify innovative agricultural practices inspired by the agroecological model on the theme of biodiversity. Next steps include implementing practices based on farmer experience on the experimental farm, and transposing successful ones to the regional network of farms in a collaborative feedback loop. First analysis show that farmers are rethinking their system to create ecological corridors for different species such as natural enemies of pests, shorebirds or game, combining functional and patrimonial aspects of biodiversity.

Both examples show that research-action projects have the potential to unlock the locks of conventional agriculture, which is overly focused on, and dangerously dependent of, habits stemming from post-war technological advances. Starting from agroecological lighthouse farms, they have the advantage of being conceived from the beginning by a core of local to regional actors. They become part of a dynamic that is embedded in a broader socio-economic and political context, often national or even supranational. Therefore, to be successful and contribute to the agroecology expansion, the bottom-up approach must be supported and relayed by policy stakeholders at a larger scale. 
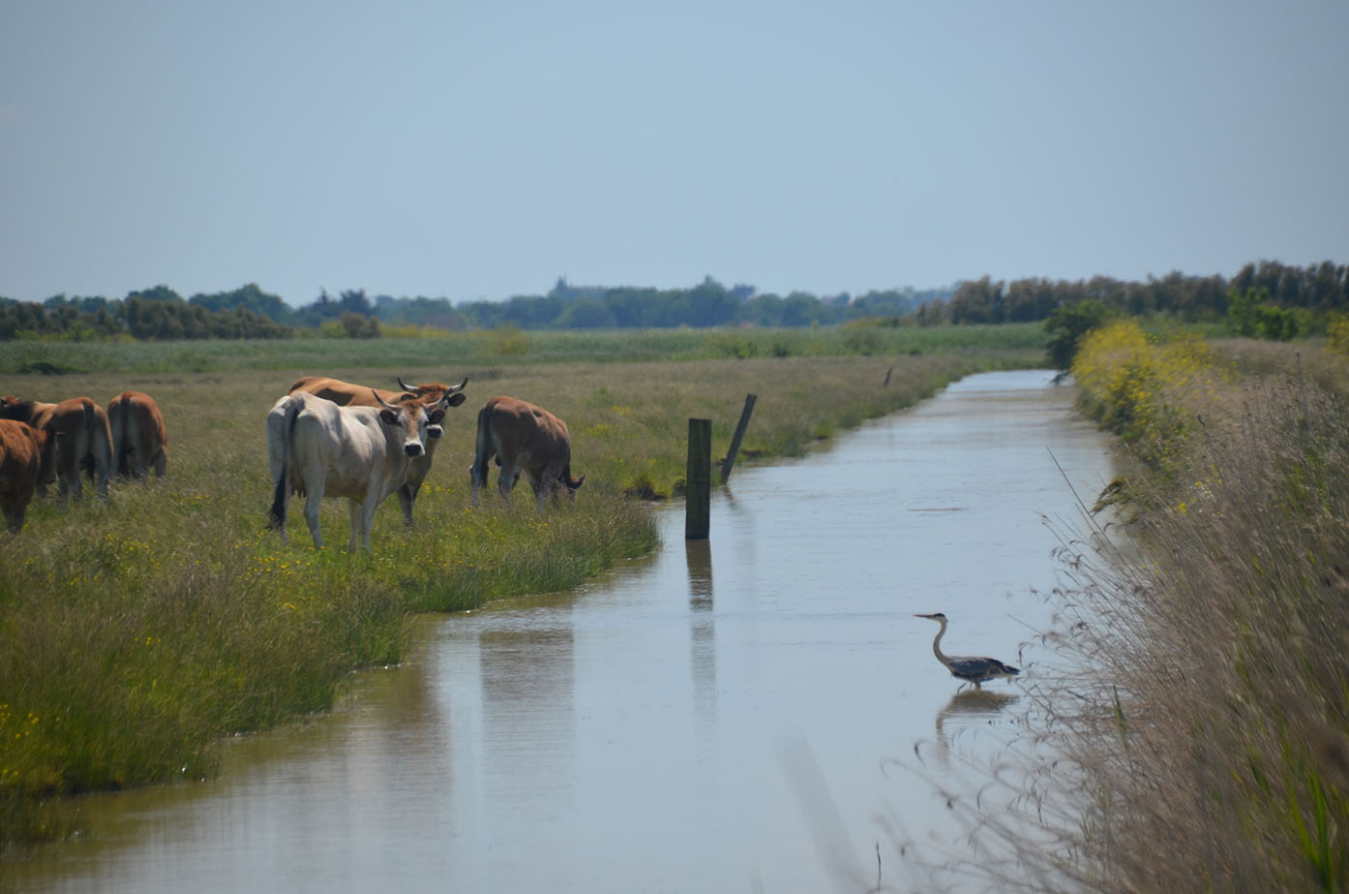

## Conclusions

One of the foundations of agroecology is the recognition that agricultural production fields are agroecosystems (Baudry et al. 2000; Vasseur et al. 2013). Though agroecosystems are often strongly simplified, monocultural, and devoted to the production of food or fibre, they pertain to the same ecological principles as other ecosystems. The functioning of these ecosystems is intrinsically dependent on biodiversity (Chapter 2). Most biodiversity issues need landscape scale consideration because of the natural tendency of any living organism to spread and colonize new habitats. Agroecosystems inserted in farmed landscapes are no exception, and their biodiversity-based functioning is embedded in a landscape context. Putting in place an agroecological approach of pest management and improve crop pollination are excellent examples (Chapter 3).

There are a couple of issues regarding the implementation of agroecology in the European agricultural landscapes. For instance, what are the most efficient agroecological management measures, and what are the measures aiming at? There may be trade-offs, e.g. between promoting measures for biodiversity conservation and supporting certain ecosystem services. Further, we do not know at this stage if measures will still be valid if agroecology is deployed at large scale – and on the long run under climate change. New knowledge needs to be acquired and new questions will arise along the process and the process itself may be questioned. For example, should the increase in agroecological farming area be clustered or dispersed, increase slowly or quickly?

The development and implementation of agroecological systems requires interdisciplinary collaboration between different branches of science. The role of landscape ecology is in the spatial targeting and in the evaluation of ecosystem service provision. This information is instrumental for formulating agricultural policies. While these policies create incentives (and dis-incentives) for the uptake, the ultimate decision makers, however, are landowners and farmers. Direct transdisciplinary collaboration with and involvement of farmers and administrators in a joint research approach can therefore accelerate the transition to agroecological landscapes.
